# Recombinant Expression and Characterization of Human and Murine ACE2: Species-Specific Activation of the Alternative Renin-Angiotensin-System

**DOI:** 10.1155/2012/428950

**Published:** 2012-02-28

**Authors:** Marko Poglitsch, Oliver Domenig, Cornelia Schwager, Stefan Stranner, Bernhard Peball, Evelyne Janzek, Bettina Wagner, Helmut Jungwirth, Hans Loibner, Manfred Schuster

**Affiliations:** ^1^APEIRON Biologics AG, Campus-Vienna-Biocenter 5, 1030 Vienna, Austria; ^2^Graz University of Technology, Rechenbauerstraße 12, 8010 Graz, Austria; ^3^University of Graz, Universitätsplatz 3, 8010 Graz, Austria

## Abstract

Angiotensin-converting enzyme 2 (ACE2) is a monocarboxypeptidase of the renin-angiotensin-system (RAS) which is known to cleave several substrates among vasoactive peptides. Its preferred substrate is Angiotensin II, which is tightly involved in the regulation of important physiological functions including fluid homeostasis and blood pressure. Ang 1–7, the main enzymatic product of ACE2, became increasingly important in the literature in recent years, as it was reported to counteract hypertensive and fibrotic actions of Angiotensin II via the MAS receptor. The functional connection of ACE2, Ang 1–7, and the MAS receptor is also referred to as the alternative axis of the RAS. In the present paper, we describe the recombinant expression and purification of human and murine ACE2 (rhACE2 and rmACE2). Furthermore, we determined the conversion rates of rhACE2 and rmACE2 for different natural peptide substrates in plasma samples and discovered species-specific differences in substrate specificities, probably leading to functional differences in the alternative axis of the RAS. In particular, conversion rates of Ang 1–10 to Ang 1–9 were found to be substantially different when applying rhACE2 or rmACE2 *in vitro*. In contrast to rhACE2, rm ACE2 is substantially less potent in transformation of Ang 1–10 to Ang 1–9.

## 1. Introduction

The classical renin-angiotensin-system (RAS) is a proteolytic cascade which is constituted by multiple enzymes and effector peptides. The cascade starts when Angiotensin I (Ang 1–10) is released from the propeptide angiotensinogen by kidney-secreted renin. The peptide metabolites produced from Ang 1–10 by a variety of proteases act as ligands for angiotensin receptors in different tissues leading to a diversified panel of physiological functions mediated by angiotensin peptides [1].

Angiotensin II (Ang 1–8) is one of the most extensively studied angiotensin peptides. It is mainly produced by the proteolytic action of angiotensin-converting enzyme (ACE) by removal of the two C-terminal amino acids from Ang 1–10. Ang 1–8 is able to bind to several cellular receptors leading to a variety of physiologic effects among different tissues and cell types [2]. Importantly, increased levels of Ang 1–8 are reported to be associated with life-threatening pathologic conditions including hypertension, congestive heart failure, chronic kidney disease, and also tumor progression [3]. Ang 1–8 was described to directly increase blood pressure and vessel permeability, to induce Na reabsorption and ROS production and excert proinflammatory and proliferative effects on various cell types [4, 5].

The disease-promoting functions of Ang 1–8 convert it to a favorable therapeutic target in the treatment of many diseases mainly by preventing its formation by low-molecular-weight compounds inhibiting appropriate enzymes of the RAS cascade. An alternative way of decreasing Ang 1–8 levels became available over the recent years and uses recombinant angiotensin-converting enzyme 2 (ACE2) to lower Ang 1–8 levels. ACE2 inactivates Ang 1–8 by clipping off one C-terminal phenylalanine [6], while Ang 1–7 is generated. Ang 1–7 is known to take over Ang 1–8 antagonistic functions by activating the MAS receptor [7–9] and therefore is thought to be the key effector peptide of the so-called alternative RAS.

Therefore, the monocarboxypeptidase ACE2 is a key activator of the alternative RAS and is critically involved in the regulation of the classical RAS, which is known to be functionally important in the vascular system and in a variety of organs [6, 10, 11]. The biological function of the RAS has been investigated in cardiovascular [12, 13], pulmonary [14], fibrotic [15], nephrologic [13], and artheriosclerotic [16] models.

Throughout all these studies the loss of ACE2 activity in knock-out variants induced pathologies which could be restored by systemic administration of the recombinant enzyme. ACE2 therefore can be regarded as one of the key players of the renin-angiotensin-system (RAS) being responsible for fluid homeostasis, blood pressure regulation, inflammatory processes, and cell proliferation. ACE2 is a membrane anchored glycoprotein which is expressed in most organs and blood vessels and recognizes multiple peptide substrates within the RAS and other peptide hormone systems. Among its substrates beside Ang 1–8, Ang 1–10, and des-Arg-bradykinin, Apelins and Dynorphins have been reported to be cleaved by ACE2 *in vitro* [17] with Ang 1–8 being the preferred substrate regarding conversion rates [18].

We recombinantly expressed both human ACE2 (rhACE2) and murine ACE2 (rmACE2) and compared their substrate conversion rates *in vitro* and in blood plasma which represents the natural compartment of enzyme action. In previously mentioned murine knock-out models, rhACE2 was frequently used to restore ACE2 activity. Despite the fact that sequence coverage between murine and human ACE2 is only 83% [19], it has been assumed that the enzyme has the same catalytic activity and function. In this work we will highlight species-specific differences between human and murine ACE2 regarding their function of keeping the balance between the classical and the alternative RAS.

## 2. Material and Methods

### 2.1. Recombinant Expression of Murine and Human ACE2

The extracellular domains of human or murine ACE2 [6] were recombinantly expressed in CHO cells under serum-free conditions. The sequence identity between rhACE2 and rmACE2 accounts to 84% which leads to minor alterations in physicochemical properties and altered patterns in posttranslational modifications, especially N-glycosylation. Both expression products were purified by sequentially performing a capture step on a DEAE-Sepharose, ammonium sulfate precipitation, followed by a purification step on a HIC-Phenyl Sepharose column and a final polishing step on a Superdex 200 gel filtration column. The purity of rhACE2 and rmACE2 was determined by high-performance liquid chromatography (HPLC) and was found to exceed 98%. The concentrations of final ACE2 preparations were determined by size-exclusion chromatography (SEC) and in line with photometric measurement at 280 nm and peak integration (OD_280_: rhACE2: *ε* = 1621 L∗mol^−1^∗cm^−1^, rmACE2: *ε* = 1750 L∗mol^−1^∗cm^−1^).

### 2.2. Native PAGE

2 *μ*g of rmACE2 and rhACE2 were applied on a precast native 3–12% gradient gel (Invitrogen). Anode buffer (50 mM Bis/Tris, 50 mM Tricine) and cathode buffer (Invitrogen NativePAGE Cathode Buffer Additive, 50 mM Bis/Tris, 50 mM Tricine) were used to run the gel. 40% glycerol, 200 mM Bis/Tris, and 200 mM Tricine were used as a loading buffer. NativeMark Unstained Protein Standard (Thermo Scientific) was used for estimation of molecular weights in Coomassie Blue-stained gels. The gel was run at 150 V for 80 min. Proteins were stained in gel using NOVEX Colloidal Blue Staining Kit according to manufacturers' recommendations.

### 2.3. SDS-PAGE

Samples were analyzed by SDS-PAGE using a 4–12% precast gradient gel (NuPage) following reductive denaturation for 5 min at 95°C. BenchMark Protein Ladder was run on the same gel to allow molecular weight estimations. The gel was run in NuPage MES SDS Running Buffer (Invitrogen) at 150 V for 80 min. In gel protein staining was performed using a NOVEX Colloidal Blue Staining Kit according to manufacturer's protocol.

### 2.4. Kinetic Assays for rmACE2 and rhACE2

Substrate specific turnover rates for rhACE2 and rmACE2 were determined by *in vitro* kinetic analysis of Ang 1–8 and Ang 1–10 cleavage followed by HPLC-based quantification of substrate and product concentrations. Enzyme reactions were started by adding a defined amount of enzyme to substrate dilutions in MES-buffer (50 mM MES, 300 mM NaCl, 10 *μ*M ZnCl_2_, 0.01% Brij-35, pH 6.5) which were previously equilibrated at 37°C. Aliquots of the reaction mixes were taken every 10 minutes and stopped by addition of 0.5 M EDTA to a final concentration of 100 mM before HPLC-based quantification of peptides.

### 2.5. HPLC-Based Quantification of Angiotensin Peptides

The concentration of peptides in enzymatic reactions was quantified by detection of peaks eluted from the HPLC column using an in-line diode array detector. Chromatography was performed by running a gradient on a reversed-phase matrix (Source 5RPC, 4.6×150 mm, 5 *μ*m) with 0.08% H_3_PO_4_ in water as mobile phase A and 40% acetonitrile in water and 0.08% H_3_PO_4_ as mobile phase B. The optical density at 280 nM was recorded inline for all eluting peaks, and peptide concentrations were calculated via calibration curves for each individual peptide.

### 2.6. *Ex Vivo* Incubation of Plasma Samples

Anticoagulated blood was collected from healthy volunteers, and plasma was separated by 10 minutes centrifugation at 3000 RCF. Following addition of 100 pg/mL recombinant human renin (Sigma) to isolated blood plasma, rmACE2 or rhACE2 was added to the samples. After 10 minutes of incubation at 37°C, in the presence or absence of Lisinopril (Sigma), samples were chilled on ice and immediately subjected to LC-MS/MS analysis.

### 2.7. RAS-Fingerprinting: LC-MS/MS Quantification of Angiotensin Peptides

Plasma samples were spiked with 100 pg/mL stable-isotope-labeled internal standards and subjected to solid-phase extraction using Sep-Pak cartridges (Waters) according to manufacturers protocol. Following elution and solvent evaporation, samples were reconstituted in 50 *μ*L 50% acetonitrile/0.1% formic acid and subjected to LC-MS/MS analysis using a reversed-phase analytical column (Luna C18, Phenomenex) using a gradient ranging from 10% acetonitrile/0.1% formic acid to 70% acetonitrile/0.1% formic acid in 9 minutes. The eluate was analyzed in line with a QTRAP-4000 mass spectrometer (AB Sciex) operated in the MRM mode using dwell times of 25 msec at a cone voltage of 4000 volts and a source temperature of 300°C. For each peptide and corresponding internal standards, two different mass transitions were measured. Angiotensin peptide concentrations were calculated by relating endogenous peptide signals to internal standard signals provided that integrated signals achieved a signal-to-noise ratio above 10. The quantification limits for individual peptides were found to range between 1 pg/mL and 5 pg/mL undiluted plasma.

## 3. Results

### 3.1. Quality Control of rmACE2 and rhACE2 Reveals a High Degree of Purity and Functional Protein Structure

**Figure 1:**
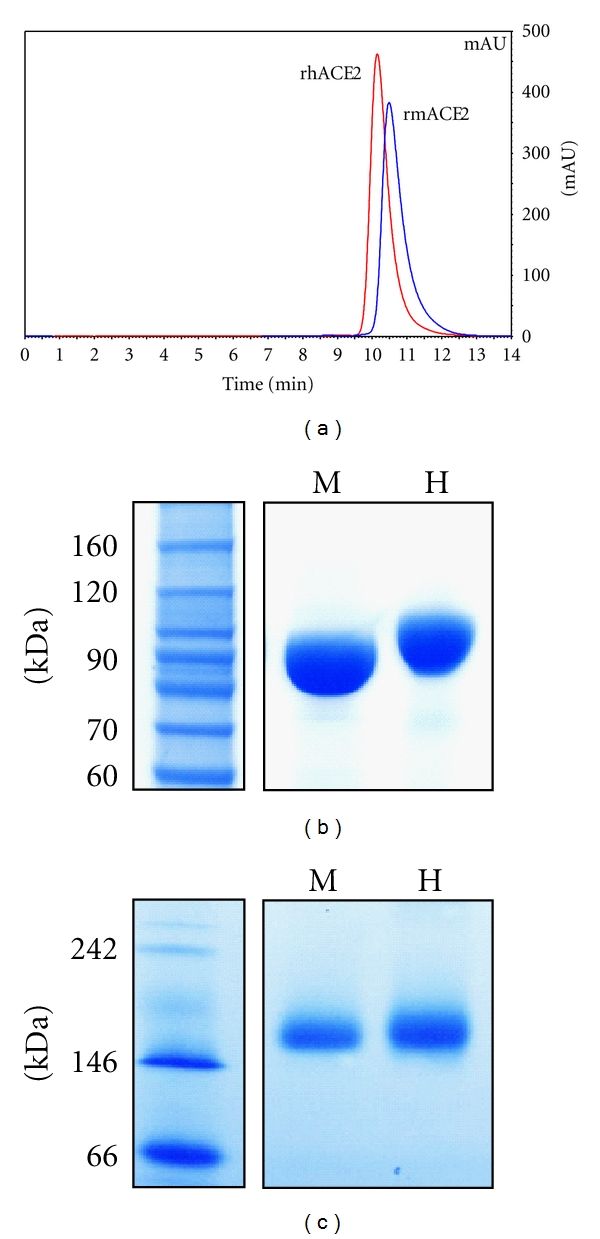
Batch quality of recombinant human and murine ACE2. (a) Equal amounts (5 *μ*g) rmACE2 and rhACE2 were analyzed by size-exclusion chromatography. The elution was continuously monitored by in-line measurement of the absorbance at 280 nm. The eluted peaks for purified rmACE2 (blue) and rhACE2 (red) are shown in the chromatogram and given in absorption units (mAU). (b) Equal amounts (7 *μ*g) of rmACE2 (m) and rhACE2 (h) were subjected to SDS-PAGE analysis followed by in-gel protein staining with Coomassie Brilliant Blue. Selected marker bands are depicted in the graph to allow the estimation of molecular weights. (c) Equal amounts (2 *μ*g) of rmACE2 (m) and rhACE2 (h) were analyzed in native PAGE followed by in-gel protein staining with Coomassie Brillant Blue as described in Section 2.

The quality of the frozen enzyme batches used for later functional analysis was analyzed regarding enzyme purity and characteristics. The investigation of rmACE2 and rhACE2 by size-exclusion chromatography revealed that no detectable contaminations were present in the enzyme preparations. The protein concentration in the enzyme batches was determined by measuring the peak absorbance inline at 280 nm, peak integration, and subsequent calculation based on the corresponding extinction coefficients. Both ACE2 variants appeared as baseline separated sharp peaks. rhACE2 and rmACE2 were found to slightly differ in retention times, pointing to a difference in their hydrodynamic molecular diameter which was found to be lower for rmACE2 (Figure 1(a)). In order to further investigate this observation, we employed SDS-PAGE analysis, revealing a mass difference under denatured conditions (Figure 1(b)), indicating the presence of additional covalent mass-increasing modifications in rhACE2. The mass shift was found to be caused by two additional glycosylation sites in the human enzyme (data not shown). According to our results, both recombinant ACE2 versions apparently occur as noncovalent homodimers in physiological solution. These findings and their possible implications will be discussed in detail elsewhere. The calculated molecular weights of monomeric rmACE2 and rhACE2 are 85.2 kDa and 85.3 kDa, respectively. RmACE2 and rhACE2 were both found to give a single band at approximately 170 kDa in native PAGE, giving evidence for a homodimer occurrence of both recombinant ACE2 versions (Figure 1(c)). These findings indicate that the recombinant enzymes, produced and purified according to our protocol, are free of contaminants and possess their natural folding and tertiary structure.

### 3.2. Recombinant ACE2 of Mouse and Human Origin Possess Diverging Turnover Rates for Natural Peptide Substrates *In Vitro*

**Figure 2:**
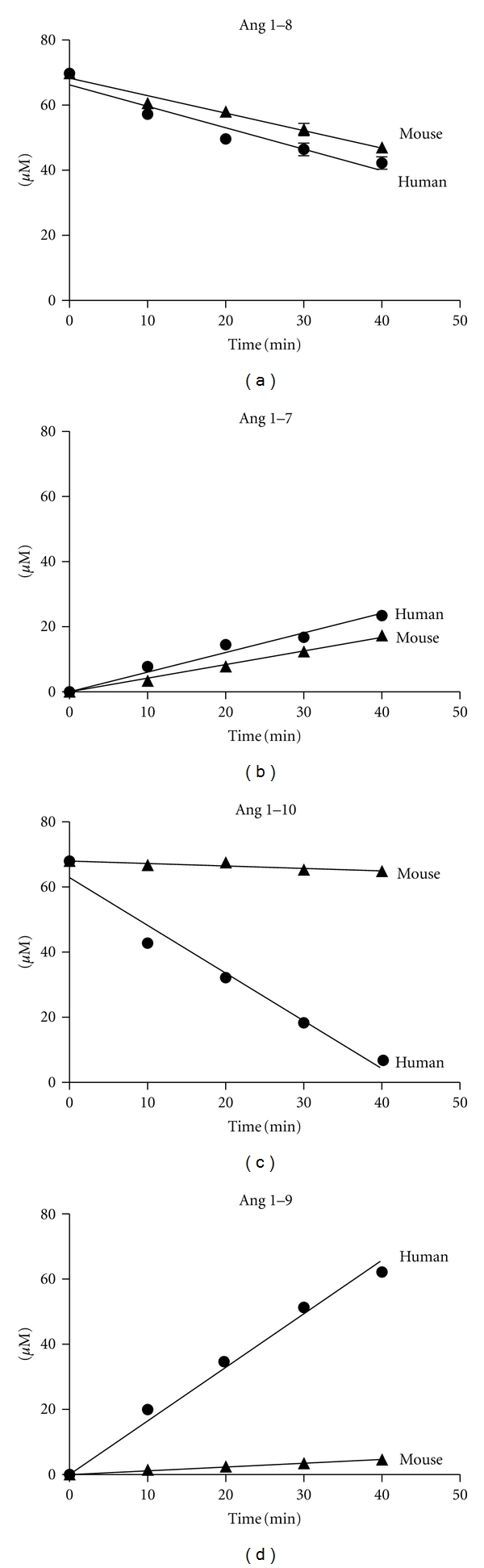
*In vitro* turnover rates of rhACE2 and rmACE2. The turnover rates for natural ACE2 substrates were determined by *in vitro* incubation with excess amounts of substrate followed by reversed-phase HPLC analysis. Therefore, 14 nM rmACE2 or rhACE2 were coincubated with 65 *μ*M Ang 1–8 (a) in MES buffer and aliquots were taken at indicated time points. The resulting time courses are shown in the graphs. The turnover rates for Ang 1–10 were determined in MES buffer containing 1.5 *μ*M rmACE2 or rhACE2 and 65 *μ*M Ang 1–10 (b) as described in Section 2. Each time point was analyzed in true triplicates and standard deviations are given in the graphs together with linear regressions of the measured values.

Based on the concentrations and purity of enzyme batches previously determined, we investigated the biological activity of rhACE2 and rmACE2 in an *in vitro* system. Therefore, we coincubated defined amounts of purified enzymes with an excess of Ang 1–8 and Ang 1–10, respectively, which represent natural substrates for ACE2. We found that rhACE2 as well as rmACE2 converted Ang 1–8 to Ang 1–7 at comparable rates (Figure 2(a)). The calculation of *k*_cat_ via the graphically determined product formation rate and substrate degradation rate revealed that the turnover number of rhACE2 for Ang 1–8 was 1.2-fold higher than that for rmACE2 (Table 1). As an alternative natural angiotensin substrate for ACE2, we also employed Ang 1–10 cleavage *in vitro*. Surprisingly, rhACE2 turned out to be much more effective in performing the cleavage of Ang 1–10 to Ang 1–9 compared to rmACE2 (Figure 2(b)). The calculation of Ang 1–10 related turnover rates for rhACE2, and rmACE2 revealed that the Ang 1–10 related *k*_cat_ for rhACE2 was 15-fold higher than that for rmACE2 (1.8 × 10^−2  ^versus 1.2 × 10^−3^ s^−1^). Furthermore, the comparison of turnover numbers for different substrates revealed that Ang 1–8 is the preferred substrate for both enzymes *in vitro* with a 42-fold higher turnover number for rhACE2 (0.77 s^−1^) and a 492-fold higher turnover number for rmACE2 (0.62 s^−1^) compared to Ang 1–10 (Table 1). These results demonstrate that human and murine ACE2 possess substantially different turnover numbers for Ang 1–10, pointing to a species-specific functional diversity of the enzyme.

### 3.3. Species-Specific Substrate Specificity of ACE2 Affects Endogenous Plasma Angiotensin Levels *Ex Vivo*

**Figure 3:**
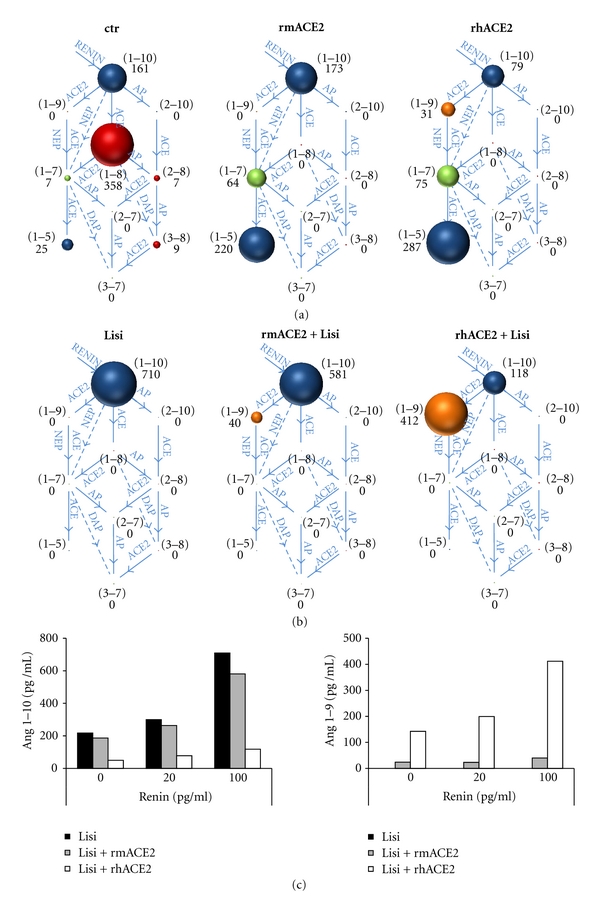
Interference of human and murine ACE2 with the endogenous RAS in human plasma. Anticoagulated plasma samples were incubated at 37°C in the presence of 100 pg/mL recombinant human renin (a, b). rhACE2 or rmACE2 were added as indicated at a final plasma concentration of 5 *μ*g/mL. RAS-Fingerprints were measured by LC-MS/MS as indicated in Section 2. Samples were incubated in the absence (a) or presence (b) of 10 *μ*M of the ACE inhibitor Lisinopril. In this figure, the diameter of the spheres reflects the concentration of the respective peptide metabolite, which is also given in pg/mL next to each individual sphere. 0 pg/mL indicates concentrations below quantification limits, which are defined by a signal-to-noise ratio below 10. Furthermore, the amino acid sequence of each angiotensin metabolite is schematically given in brackets beside the corresponding sphere. The sequence annotation is based on the decapeptide Angiotensin I (1–10) which is N- or C-terminally cleaved by the indicated proteases. Proteases are illustrated by arrows connecting their substrate and product. AP: aminopeptidases; NEP: neutral endopeptidase; DAP: di-aminopeptidase; ACE: angiotensin-converting enzyme. (c) Indicated concentrations of recombinant human renin were added to Lisinopril-treated plasma samples. Control samples (black bars), rmACE2- (grey bars), and rhACE2- (white bars) treated samples were subjected to RAS-Fingerprinting, and resulting concentrations for Ang 1–10 (left) and Ang 1–9 (right) are given in pg/mL.

In order to investigate the substrate specificity of rhACE2 and rmACE2 under physiologic conditions, we assessed the impact of the two enzymes on the human RAS in blood plasma. Therefore, we simulated a pathological hyperactivated RAS by addition of recombinant human renin to anticoagulated human blood plasma. Going in line with our previous *in vitro* findings, the addition of rhACE2 or rmACE2 to *ex vivo* incubated plasma samples revealed that both enzymes effectively degraded Ang 1–8 to yield Ang 1–7 and Ang 1–5 when compared to the enzyme-free control sample (Figure 3(a)). Although the plasma concentration of Ang 1–10 was only 161 pg/mL (124 pM), a concentration of 5 *μ*g/mL (58,8 nM) rhACE2 was found to efficiently convert Ang 1–10 to Ang 1–9, as indicated by the peptide levels depicted in the RAS-Fingerprints (Figure 3(a), right). In contrast to rhACE2, rmACE2 was unable to decrease Ang 1–10 concentrations in plasma and failed to induce detectable Ang 1–9 levels (Figure 3(a), middle). Of note, the increase of Ang 1–7 and Ang 1–5 in the presence of rhACE2 was even more prominent, due to this second pathway of Ang 1–7 production via Ang 1–9, which was selectively supported only by rhACE2.

As* in vitro* experiments revealed that rmACE2 was capable of converting Ang 1–10 to Ang 1–9, although to a much lower extent compared to rhACE2, we further investigated the capability of rmACE2 for Ang 1–9 formation in human plasma at increased Ang 1–10 concentrations. We added the ACE-inhibitor Lisinopril to our *ex vivo* setting, in order to prevent ACE-mediated degradation of ACE2-produced Ang 1–9 and to increase Ang 1–10 levels by preventing its degradation by endogenous ACE. The presence of Lisinopril led to significantly increased Ang 1–10 peptide levels compared to untreated control samples (710 pg/mL versus 161 pg/mL) (Figures 3(a) and 3(b) left). Comparison of rhACE2 and rmACE2 activities in Lisinopril-treated complete human plasma revealed that rhACE2 effectively converts large amounts of Ang 1–10 to Ang 1–9 in the physiological matrix while rmACE2 was found to be much less effective in catalysing this reaction (Figure 3(b)).

Interestingly, Lisinopril was not able to increase Ang 1–7 concentrations in our experimental settings, which was in contrast to several published reports. No Ang 1–7 was detectable in plasma samples incubated with Lisinopril in the absence or presence of rhACE2 or rmACE2 (Figure 3(b)) meaning that the concentration was below the quantification limit of 2 pg/mL plasma. For further investigation of these surprising results, the experiment was repeated for rhACE2 in whole blood instead of plasma. Whole blood incubations gave similar results as previously reported by other groups, showing an increase of Ang 1–7 concentrations in control and rhACE2 samples in response to Lisinopril (see Supplementary Figure 1 available online at doi:10.1155/2012/428950).

For further investigation of rmACE2 and rhACE2 substrate specificities, different states of RAS activity were simulated by addition of lower amounts of recombinant human renin in the presence of Lisinopril, confirming our findings about strongly diverging conversion rates for Ang 1–10 between rhACE2 and rmACE2 in a substrate concentration-dependent manner (Figure 3(c)). These results demonstrate that Ang 1–10 serves as a natural substrate for rhACE2 which is efficiently processed under physiological conditions. In contrast to that, rmACE2 is much less effective regarding this catalytic conversion, strongly supporting a species-specific role of ACE2 in the activation of the alternative RAS pathway.

## 4. Discussion

We expressed and purified both rhACE2 and rmACE2 in CHO cells under serum-free conditions. Both cell lines were stably secreting high levels of recombinant proteins for at least two months of roller bottle cultivation. The quality of the expression products did not change from early to the latest passages. Both rhACE2 and rmACE2 appeared as stable homo-dimers, while we did not identify monomeric or other multimeric forms. We evaluated the quality of the enzyme preparations by multiple methods including HPLC, SEC, SDS-PAGE, and native PAGE which all confirmed the purity of the final products and their homo-dimeric tertiary structure (Figure 1). Despite the similarity of the calculated molecular weights for human and murine monomers, surprisingly high mass differences between rhACE2 and rmACE2 were observed in SEC and could be finally identified to be caused by species-specific sequence variations which lead to a different number in N-glycosylation sites in human and murine ACE2.

ACE2 is known to cleave a variety of peptide substrates *in vitro* [18], which are involved in a broad panel of physiological functions [17]. Based on our findings about the differences in tertiary structure between rhACE2 and rmACE2, we hypothesized that the well-known sequence diversity between the two species might have an impact on the functional characteristics of the enzymes. Therefore, we assessed the turnover rates for rhACE2 and rmACE2 for two natural and physiologically important substrates (Ang 1–10 and Ang 1–8) in a well-defined *in vitro* model system (Figure 2). We selected these substrates for ACE2 characterization because of their functional importance in maintaining RAS peptide levels. It has been described previously that the angiotensin peptides Ang 1–8 and Ang 1–10 are cleaved by ACE2 *in vitro* [6]. As the conversion rates of ACE2 for Ang 1–10 were reported to be substantially slower than those for Ang 1–8, this enzyme reaction was supposed to take over a minor role in the formation of Ang 1–7 than the direct production by Ang 1–8 cleavage. We could confirm previous findings regarding substrate preferences and found a 42-fold higher turnover number for Ang 1–8 compared to Ang 1–10 when cleaved by rhACE2 (Table 1). Interestingly, our values for *k*_cat_ were lower compared to previous publications which might have been caused by differences in the employed experimental settings, in particular because of different buffer systems. Nevertheless, the qualitative conclusions were comparable and internally controlled. While showing comparable turnover rates for Ang 1–8, the Ang 1–10-related turnover rate for rmACE2 was found to be only 7% of the respective rate for rhACE2. This fundamental difference in the substrate conversion rates of the two enzymes might also have substantial impact on the regulation of the RAS under physiologic conditions in the two different species.

Unfortunately, only limited conclusions about physiological consequences can be drawn out of *in vitro* experiments. An important feature of the physiologic conditions in blood plasma is that all RAS enzymes except renin are present in excess compared to their substrates, which is the exact opposite of the *in vitro* situation. Although *in vitro* investigations are very useful for the comparison of enzyme characteristics in one and the same model system, they tell us very little about the *in vivo* situation.

Therefore, we developed an *ex vivo* experimental setup which allowed us to investigate the enzymatic function of the two recombinant enzymes in their physiological environment, with their natural substrates being present at picomolar concentrations. We would like to point out that these *ex vivo* conditions reflect the human *in vivo* plasma conditions regarding circulating enzyme concentrations after systemic administration of rhACE2 (data not shown). Although *ex vivo* incubations are very reproducible and reflect an integrated picture of soluble enzyme activities throughout the RAS in undiluted plasma, the angiotensin peptide concentrations are clearly higher in *ex vivo* incubated plasma samples, which might be caused by a lack of the peptide flow towards organs or endothelial surfaces *ex vivo*.

We investigated the RAS in these samples by means of a newly developed LC-MS/MS method, which allows the quantification of multiple angiotensin metabolites simultaneously in one single sample of blood plasma. The obtained RAS-Fingerprints revealed that, in contrast to rmACE2, rhACE2 is capable to generate Ang 1–9 from Ang 1–10 at physiologic peptide concentrations (Figure 3). This activity even more gains importance in the presence of the ACE-inhibitor Lisinopril, which blocks the formation of Ang 1–8. Under latter conditions, large amounts of Ang 1–9 are generated in the plasma samples by rhACE2, while rmACE2 is much less effective in its formation of Ang 1–9 from Ang 1–10.

Although significant amounts of Ang 1–10 and Ang 1–9 were present in samples treated with Lisinopril alone or in combination with rhACE2, no Ang 1–7 could be detected in these samples. These findings were in contrast to previously published reports on Ang 1–7 accumulating effects of ACE inhibitors *in vivo *[20, 21]. In our experimental setting, we employed heparinized blood plasma as a sample matrix for ACE2 characterization which is reflecting *in vivo* conditions very well. However, plasma lacks all blood cells which might carry receptors and angiotensin peptide converting enzymes being able to affect angiotensin peptide concentrations *in vivo*. Comparison of plasma and whole blood samples revealed that Lisinopril-induced Ang 1–7 accumulation is strictly dependent on blood cell-associated angiotensin peptide converting enzymes, as it was exclusively observed in whole blood *ex vivo* incubations (Figure 3(b), Supplementary Figure 1). As neutral endopeptidase (NEP, CD10) is known to be expressed on the cell surface of leukocytes [22, 23] and that it is able to convert Ang 1–10 and Ang 1–9 to Ang 1–7 *in vitro* [18], NEP is very likely to be responsible for Ang 1–7 accumulation also *in vivo,* especially in the presence of ACE inhibitors which block the formation of Ang 1–8 which is an important precursor for Ang 1–7.

In human plasma, the ACE2-mediated formation of Ang 1–9 from Ang 1–10 represents a significant route of establishment of the alternative RAS. This may be, for example, of particular importance *in vivo*, when ACE inhibitors are used for antihypertensive treatment. As ACE2 is primarily expressed as a membrane-attached enzyme in several organs [24], the local production of Ang 1–9 from Ang 1–10 which is increased when ACE inhibitors are present, might become an important mechanism of action for ACE inhibitors action *in vivo in humans*. In addition to Ang 1–7, also Ang 1–9 has been reported to possess protective effects in cardiovascular disease models [25]. These mechanistic considerations seem to be of particular importance in humans, while murine model systems for the investigation of ACE inhibitor efficacy might be reconsidered in respect to the species-specific lack of Ang 1–10 cleavage by murine ACE2.

Altogether, our findings describe important species-specific differences in the fine specificity of ACE2. Thus, the murine RAS is likely to function differently when compared to its human counterpart. Furthermore, our data point to the importance of further investigations and improved understanding of the human RAS, while data generated in murine model systems might be partially reconsidered in respect to different enzyme properties. Deciphering the functional characteristics of the human RAS using new analytical possibilities reveals previously invisible features of the system. The future generation of human-derived data describing RAS function in health and disease will pave the way for new concepts of therapeutic manipulations of the system which are more specifically designed for application in humans.

## Figures and Tables

**Table 1:** Enzymatic properties of rhACE2 and rmACE2. The substrate turnover rates and product formation rates were determined for the kinetic experiments in Figure 2. Therefore, the slope of the linear regressions across all data points was determined for both substrate-product pairs and both enzymes. The mean of the absolute values for the substrate degradation rates and product formation rates for each enzyme were related to the molar concentration of rhACE2 or rmACE2. The resulting values for *k*_cat_ are given in the table.

	*k*_cat_ [s^−1^]	
	rhACE2	rmACE2

Ang 1–8 ≥ Ang 1–7	7.7 × 10^−1^	6.2 × 10^−1^
Ang 1–10 ≥ Ang 1–9	1.8 × 10^−2^	1.3 × 10^−3^
